# The Art of Muscle Biopsy in the New Genetic Era: A Narrative Review

**Published:** 2019

**Authors:** Yalda NILIPOUR

**Affiliations:** 1 Pediatric Pathology Research Center, Research Institute for Children’s Health, Mofid Children Hospital, School of Medicine, Shahid Beheshti University of Medical Sciences, Tehran, Iran.; 2 Mofid Children Hospital, School of Medicine, Shahid Beheshti University of Medical Sciences, Tehran, Iran, Email: yalnil@yahoo.com

**Keywords:** Muscle biopsy, Myopathology, Indication, Diagnostic algorithms, WES

## Abstract

Muscle biopsy has been practiced as a well-established part of paraclinical workup in patients with neuromuscular diseases since late 1960s. In this narrative review paper, the role of some pre and post muscle biopsy factors and their importance in achieving the best diagnostic results will be explained. Considering the new advances in diagnostic molecular techniques and the availability of local standard myopathology laboratory as well as the presence of a dedicated myopathologist, the indications of muscle biopsy in four major types of neuromuscular diseases will be shortly reviewed. Moreover, indications of muscle biopsy in four major types of neuromuscular diseases will be shortly discussed based on literature review of recent published diagnostic algorithms and our 11 years’ experience of performing about 4000 muscle biopsies cases in the only standard referral diagnostic center for muscle biopsy in Iran. Although diagnostic algorithms of some muscle diseases have been changed based on recent advances in biochemical and molecular diagnostic techniques, still muscle biopsy continues to play a major role in the diagnosis and management of variety of neuromuscular disorders and has proved to be a preferable diagnostic procedure by some neuromuscular specialists for the cases who can benefit from rapid therapeutic management. The application of diagnostic algorithms should be practiced in accordance with geographic distribution of the diseases, the availability of diagnostic techniques and the presence of specialists in each center considering the local insurance coverage and the cost to be paid by the patient in every center.

## Introduction

Muscle biopsy has been considered as an essential part of paraclinical workup of patients with myopathic and neuropathic (Neurogenic) diseases alongside with physical examination, laboratory testing, electromyography and molecular investigations. The credit of morphologic study of muscle biopsy is historically given to the French Neurologist Guillaume-Benjamin-Amand Duchenne (1). Generally based on histologic findings of muscle biopsy the physician can determine if a muscle is normal or abnormal but even a normal muscle biopsy does not exclude neuromuscular diseases. Histopathologic findings of a muscle biopsy could be either specific for diagnosis or nonspecific. Therefore, all histopathological findings should be interpreted in the light of each patient's phenotype. 

Muscle biopsy is usually not the first diagnostic test requested when the clinical phenotype of a myopathic patient is clear and the molecular diagnosis is straight forward. For example, ordering specific genetic study of D4Z4 gene and Multiplex Ligation-dependent Probe Amplification (MLPA) of dystrophin gene are the primary requested evaluation tests for a patient with typical phenotype of Fascioscapulohumeral muscular dystrophy (FSHD) and Duchenne muscular dystrophy (DMD) respectively. Generally, a physician approaches a disease in a fairly certain way so that a correct diagnosis would not likely to be reached in a less expensive and less invasive manner. Along with great recent advances in the techniques of molecular diagnosis such as next-generation sequencing (NGS) and the introduction of new serologic markers in recent years, the diagnostic strategy of some neuromuscular diseases has been modified and the position of the applicability of muscle biopsy in the diagnostic algorithms has been modified and even questioned by some clinicians for certain types of neuromuscular diseases.

**Muscle biopsy ordering and procedure**

Muscle biopsy has been historically considered as a minimally invasive diagnostic test in the hands of neuromuscular specialists, neurologists and rheumatologists. When the differential diagnosis is wide, a muscle biopsy is recommended in the early stages of care in order not to lose time in treatable diseases. There are also some factors limiting the diagnosis of muscle diseases only based on genetic testing. Basically, novel diseases and novel genes could not be easily diagnosed only by molecular techniques without any functional evidence. Moreover, the correct diagnosis of phenotypical variants of non-genetic diseases is sometimes challenging. For example, by omitting muscle biopsy from diagnostic algorithms of inflammatory myopathies, the diagnosis of adermatopathic juvenile dermatomyositis "dermatomyositis sine dermatitis" will be easily missed even with the most sophisticated molecular diagnostic techniques (Case 1). 

**Case 1**

**Figure 1:**
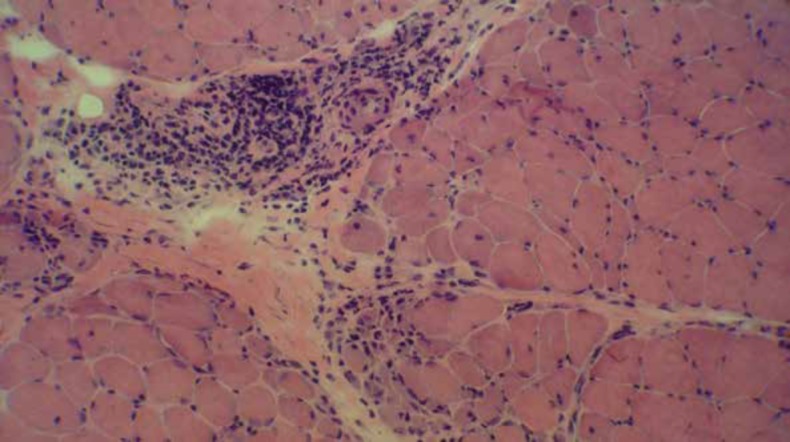
Perifascicular atrophy with perivascular perimysial chronic inflammation typical for dermatomyositis (H&E on frozen section x200)

A 5-year-old boy adopted from unknown parents presenting with difficulty of rising from floor and climbing stairs for a couple of months with no skin lesion. His serum CK reported to be about 2000. EMG study revealed myopathic pattern. MLPA study of Dystrophin gene presented no mutation. Then WES was requested in which no pathogenic mutation was found. Finally, the little adopted boy who was going to lose his new family by clinical impression of progressive hereditary muscle disease underwent muscle biopsy. The diagnosis of “juvenile dermatomyositis sine dermatitis” was obtained by muscle biopsy (Figure 1). Immunosuppressive therapy started for him right after biopsy result. In his next 2-months follow up he had normal physical examination.

**Case 2**

A 34-year-old woman presented to emergency room with loss of conciseness, generalized muscle weakness and severe respiratory distress. She had profound metabolic acidosis and went under ventilator. Her biochemical assays showed normal results. The diagnosis of "lipid storage myopathy" was reported to the physician on the same day of muscle biopsy (Figure 2). Treatment with a cocktail of high dose Riboflavin, Carnitine and Q10 supplement was started for her. She showed dramatic response and the muscle power of all tested muscles were normalized within one month follow up.

**Figure 2a:**
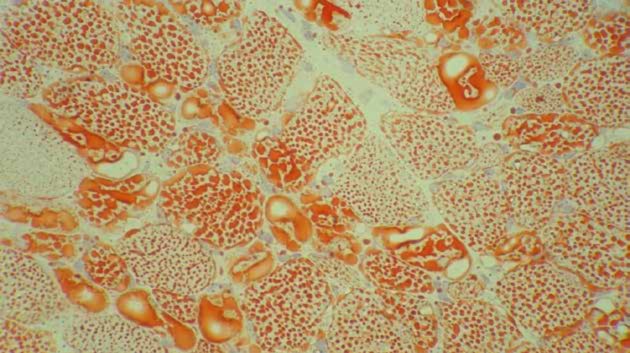
Vacuolar myopathy with variable sized clear cytoplasmic and subsarcolemmal vacuoles (H&E on frozen section x400)

**Figure 2b:**
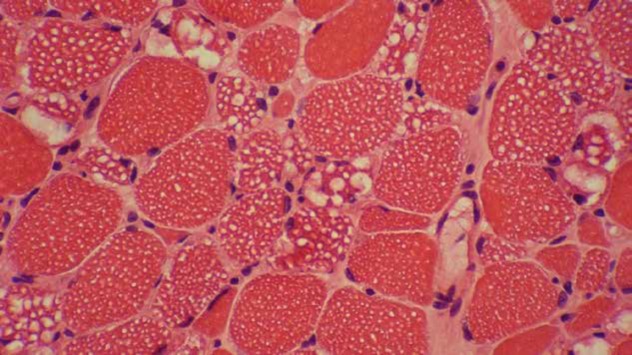
Prominent lipid excess in muscle fibers and cytoplasmic vacuoles (Oil red O stain on frozen section x400)

Although muscle biopsy is not a complex procedure it should be asked and performed by a physician who is well aware of neuromuscular diseases and their mimics (2). Based on the results of performing about 4000 muscle biopsy cases during the past 11 years we can make sure that receiving the best results from this procedure needs good teamwork of several specialists, including an experienced clinician and electrophysiologist, a specially trained surgeon, and a dedicated myopathologist. 

In order to obtain the optimum diagnostic findings from muscle biopsy, a full clinical assessment of the patient is required along with meticulous selection of the time and site of muscle biopsy. Knowledge of distribution of muscle involvement in different myopathies is essential in choosing an appropriate candidate for muscle biopsy and selection of the best site for biopsy. Choosing the right site of biopsy is a big challenge in some cases. By performing routine muscle biopsy from vastus lateralis muscle, diagnosis of quadriceps sparing myopathies will be impossible by muscle biopsy. A muscle with Medical Research Council (MRC) grade 4/5 strength on physical examination is usually informative and a proper site for muscle biopsy (2). When upper limbs are affected alongside with lower limbs we usually choose non-dominant upper limb, in order not to complicate the daily life activities of the patient after biopsy. We need to make sure that the selected muscle has not been recently subjected to electromyography (EMG) needling or a previous biopsy or injury. In cases with selective muscle involvement when the affected muscles are completely gone and the remaining muscles are spared, choosing the right site for muscle biopsy is a big challenge. Eventually, biopsy of the end-stage muscles should be avoided.

A comprehensive EMG report will help to choose the proper site of muscle biopsy. The recent application of muscle imaging, especially muscle magnetic resonance imaging (MRI), has been also a great help to select the appropriate site of muscle biopsy in difficult cases.

Normal muscle force does not exclude indication of muscle biopsy. The chance of obtaining a specific diagnosis in asymptomatic hyperCKemia has been reported to range from 7% to 70% of cases based on different studies (3). Traditionally, quadriceps, deltoid and biceps are chosen for biopsy in cases of proximal weakness and gastrocnemius, tibialis anterior and brachioradialis are appropriate choices for distal limb weakness (2). 

The size of muscle biopsy sample could be varied based on considered differential diagnosis and the planned testing before biopsy. In the case of requiring tissue only for IHC study of a specific protein loss, even a small maloriented markedly atrophic sample could be sufficient for a definite diagnosis. On the other hand, a barely affected muscle is also appropriate for biopsy in cases of searching for expression of specific muscle associated proteins.

Joint meetings and planning before muscle biopsy ordering is essential for the assurance quality of the results. If muscle biopsy procedure is not performed by a neurologist or the neuropathologist himself, the ordering physician should supervise the details of sampling and delivery of the muscle tissue to prevent any possible artifacts in order to get the best diagnostic results from the procedure. In addition, the logistical issues of muscle tissue handling have to be solved between clinicians and the myopathology laboratory prior to performing the biopsy to avoid possible need of biopsy repetition due to inappropriate sample quality or formalin fixation of the muscle specimen (2, 4). 

Generally, biopsy from almost all other body organs needs only formalin fixation as a routine procedure, but standard muscle biopsy requires freezing a fresh specimen in liquid phase of Isopentane, cooled in liquid nitrogen. This protocol provides frozen section specimen for a good morphology and enzyme histochemical and immunohistochemical studies. No matter how experienced the pathologist is, a formalin-fixed muscle sample, even oriented or fixed on wood, will not provide a definite diagnosis (5). The only possible exception is the classic dermatomyositis with typical perifascicular atrophy sometimes recognized even on a non-oriented, formalin-fixed needle biopsy specimen.

The development of percutaneous needle muscle biopsy technique by Duchenne in 1961 and later by Bergstrom in 1962 provided an alternative to open biopsy. It is still routinely used in some centers (6). However, open biopsy under local anesthesia is our preferable technique of choice as an outpatient minor surgical procedure. It is well tolerated and the size and the quality of the sample are guaranteed. Based on our personal experience the risk of hematoma or infection is also very low (7).

Multi-disciplinary review sessions between neurologist, myopathologist and even a genetic specialist are helpful in clinicopathologic correlations of biopsy findings. The knowledge of terminology of histologic findings by clinicians will facilitate the interpretation of histologic findings for the right diagnosis of the patient. There is no established turnaround time for the final muscle biopsy pathology report along with histochemical and immunohistochemical stains (8). In our clinical practice based on the first frozen H&E slide findings a short hand writing diagnostic report will be communicated to our colleagues in cases when immediate therapeutic intervention is required. 

**Muscle biopsy in four major categories of muscle diseases**

In what follows, based on recent published diagnostic algorithms and our 11 years’ experience of performing about 4000 muscle biopsies cases in the only standard referral diagnostic center for muscle biopsy in Iran, the indications of muscle biopsy in four major types of neuromuscular diseases will be shortly reviewed. As it is outside of the scope of this narrative review article to go through all the details the tips for the most important and common diagnoses will be briefly mentioned.

**Muscle biopsy in metabolic myopathies**

Metabolic myopathies are a heterogeneous group of genetic disorders that may be present at any age. They are classified based on their biochemical abnormalities as disorders of carbohydrate or lipid metabolisms as well as mitochondrial disorders. Traditionally it is preferred to clinically verify which one is the most likely affected biochemical pathway then the diagnostic tool is selected. The diagnostic algorithms may start with targeted analysis of more common mutated genes. Presently the new diagnostic approaches are trending to start with broad genetic testing such as whole-exome sequencing (WES) (3, 9).

The new algorithms must be applied and modified in each center based on their geographic distribution of different neuromuscular diseases and the availability of standard myopathology laboratory. For example, in our center, a muscle biopsy is ordered in patients suspected of having metabolic myopathies before any molecular study because early diagnosis is important to start treatment as soon as possible. Lipid storage myopathies are fairly common in our region and the patients' life can be saved by starting early medication right after biopsy results. Early treatment in these patients can prevent fatal myoencephalopathy crisis (Case 2)

 We also perform muscle biopsy at the same time with Dried blood spot (DBS) test for hypotonic infants or a myopathic patient suspected of Pompe' disease as we can provide Hematoxylin and eosin (H&E), periodic acid-Schiff (PAS) and Oil red O stains of frozen muscle sample at the same working day of muscle biopsy. This procedure allows the physician to start the required treatment as soon as possible while waiting for DBS results.

There are certain types of myopathies that may present with pseudometabolic pattern. For example, sarcoglycanopathy could present with episodes of rhabdomyolysis. In these cases, muscle biopsy is also important for definite diagnosis (9-11). 

Muscle biopsy remains the gold standard for diagnosis of mitochondrial diseases, especially for mtDNA diseases, because of heteroplasmy and tissue-specific mutations (12).

**Muscle biopsy in inflammatory myopathies**

Inflammatory myopathies (IM) are heterogeneous nonhereditary disorders. The main types include polymyositis (PM), dermatomyositis (DM), necrotizing myopathy (NM), overlap syndrome with myositis (OM) like anti-synthetase syndrome (ASS), and inclusion body myositis (IBM). The correct diagnosis of IMs requires full assessment of symptoms of the patient including detailed medical history, pattern of muscle weakness, paraclinical tests such as muscle enzymes, EMG and new myositis specific auto-antibodies (MSA) as well as detailed histopathological workup of a muscle biopsy (13). 

Except for classical dermatomyositis the exact diagnosis of IM is challenging these days and some novel diagnostic criteria have been proposed (14-16).

IHC expression of major histocompatibility antigen type 1 (MHC-1) on non-necrotic muscle fibers could be seen in IMs, even in the absence of inflammatory cells infiltration. Although this expression has also been reported in cases of dysferlinopathy, DMD and FSHD (5). New IHC markers are proposed for the diagnosis of difficult cases. In cases of DM with typical skin rashes but no perifascicular atrophy, sarcoplasmic myxovirus resistance protein A (MxA) expression is reported to be more sensitive than capillary membrane attack complex (MAC) deposition and is claimed to be a more sensitive marker for diagnosis of DM than other classical pathological features (17-19). Immunostaining with p62 antibody, although is not specific for IBM, could facilitate distinguishing IBM from DM and PM (5). 

In cases of IM the best result can be achieved by detailed morphological evaluation of muscle biopsies in combination with clinical workup and serologic analysis. Utilization of MSA is claimed to be crucial for the correct diagnosis of IMs and it provides prognostic information and predicts treatment response, although in about one-third of the IMs no MSA will be found (13, 16, 17, 19, 20). In a case of progressive weakness not responding to treatment, it is not illogical to repeat muscle biopsy.

**Muscle biopsy in muscular dystrophies**

Muscular dystrophies are hereditary progressive muscle diseases due to mutations of different proteins localize to the sarcolemma, sarcomere, myonuclei, basement membrane and/or extracellular matrix (5, 11). When the phenotype is typical for a specific diagnosis, the physician will ask for the least expensive and the less invasive diagnostic tests. For example, MLPA study of dystrophin gene is the first requested study for a Duchenne-like phenotype boy who has high Creatine Kinase (CK). 

IHC expression study of some proteins on a muscle biopsy specimen can identify the defect of the proteins responsible for different types of muscular dystrophies. It should be complimentary to conventional histology and a panel of histochemical study but it should not be interpreted alone. Prior to the introduction of NGS technique, IHC study results used to be essential guidance for genetic studies of muscular dystrophies but presently it is mostly recommended for the confirmation of the genetic findings and plays a role as a functional study. 

Although the advances in molecular diagnostic techniques have limited the role of muscle biopsy in the diagnosis of DMD and BMD, muscle biopsy is still relatively a common requested test for Duchenne-like phenotype young boys who have negative MLPA study of Dystrophin gene. Loss of immunohistochemical or immunofluorescent expression of dystrophin antibodies on a frozen muscle sample is still a gold standard for the diagnosis of dystrophinopathy and the carriers. It is crucial to keep in mind that current methods of commercial genetic analysis can detect only up to 93% to 96% of mutations (REF). The remaining genetically undiagnosed cases are subject to have muscle-derived mRNA to clarify the related genetic abnormality (21). The data from our unpublished personal experience revealed that no mutation was found by WES in 20% of MLPA negative approved DMD cases by muscle biopsy.

WES or a panel of myopathic genes has been suggested before muscle biopsy in international diagnostic algorithms for a patient suspected of LGMD (22). In an international project of molecular analyzing of 450 muscle disease genes by NGS technique, no pathologic variant has been detected in 50% of 1001 patients affected by limb-girdle weakness from 21 countries including Iran (23). IHC study of muscle proteins on a muscle biopsy sample has a crucial role in the diagnosis of LGMD patients in whom WES fails to identify the pathogenic mutation or reveals genetic variants of unknown significance (VUS). Our limited study of LGMDs revealed that beta Sarcoglycanopathy is the most common type of sarcoglycanopathy in Iran (24). We recommend to start with a muscle biopsy in suspected cases of sarcoglycanopathies and then the diagnosis of sarcoglycanopathy can be confirmed by MLPA study of sarcoglycan genes. Accordingly, a non-expensive definite diagnosis can be provided for most cases of sarcoglycanopathy in Iran.

**Muscle biopsy in structural myopathies**

Congenital myopathies are a clinically, molecularly and morphologically a heterogeneous group of muscle diseases and are mainly due to defects in sarcomeric proteins. Unlike its label the presentation of symptoms could be presented later in adulthood. Classification of congenital myopathies is still based on characteristics of histological findings of muscle biopsy. The most common types are core myopathies, nemalin myopathy, centronuclear myopathy and congenital fiber-type disproportion. Since these histological findings are not gene-specific and there are considerable phenotypical variations with each histological hallmark, and considering recent identification of multiple new genes, a new classification of congenital myopathies based on responsible mutated genes has been proposed. Presently, mutations in more than 20 genes causing congenital myopathies have been identified (25, 26).

Although clinical diagnosis of congenital myopathy is not difficult, reaching a confirmed genetic diagnosis is not always easy. In our region, WES provides a relatively expensive but a fast diagnosis. The presence of the largest genes such as RYR1, TTN and NEB in the list of genes responsible of congenital myopathies makes the interpretation of the molecular findings difficult and hampers reaching a definite diagnosis in many cases. Moreover, due to the rarity of congenital myopathies we are usually faced with multiple VUSs, new mutations and even new genes. Definite diagnosis of these cases is subject to muscle biopsy for functional evidence. Reporting a new mutation in a patient with an unusual phenotype of a known congenital myopathy is also challenging (25, 26).

Recent WES testing facilitates diagnosis of congenital myopathies but it is still a big challenge in about half of the patients (27-29). Most recent diagnostic approaches for patients suspected of suffering from congenital myopathy requires integration of data from genetic testing, muscle biopsy and/or muscle imaging for the definite diagnosis (5, 22, 25, 26).

## Discussion

As it was nicely proposed in a book review of the first edition of "Pathology of Skeletal Muscle" "Muscle biopsy is an art" (30). When a muscle biopsy is requested, we must make sure that the highest quality is achieved to provide the correct diagnostic information. There are many pre and post important muscle biopsy factors to be considered in order to achieve the best diagnostic results from a muscle biopsy and multidisciplinary meetings will guarantee the correct interpretation of histologic findings to achieve the best results to the benefit of the patient. Although a major limiting factor of muscle biopsy is its invasiveness still it is not considered as an absolute barrier to request muscle biopsy, because it is minimally invasive and easily tolerated by the patient with very low rate of complications (7). Moreover, muscle biopsy provides invaluable materials for functional studies, biobanking, ultrastructural analysis, understanding the pathophysiology of diseases, RNA sequencing, and recent treatment researches (26).

The crucial role of muscle biopsy in the diagnosis of some neuromuscular diseases has been limited by WES analysis. Although molecular diagnosis is important for genetic counseling and prognosis of patients, there are still multiple issues in the diagnosis of neuromuscular diseases only based on molecular testing. The presence of non-genetic acquired diseases, identification of new mutations of known genes especially with unusual phenotypes, discovery of new genes, recognition of new diseases and limitations of genomic data in certain populations or an adopted child with unknown parents are all restrictive factors for the diagnosis of muscle diseases solely based on genetic testing. 

**In Conclusion, **the application of diagnostic algorithms should be practiced in accordance with the geographic distribution of the prevalence of different diseases, availability of diagnostic techniques and the presence of specialists in each center considering the local insurance coverage and the cost to be paid by each patient. In our modern genetic era muscle biopsy still continues to play a crucial role in the diagnosis of certain types of neuromuscular diseases either as a gold standard or as a complementary method to the genetic and serologic studies and still remains a preference method of diagnosis by many neuromuscular specialists who work on rare diseases. In a proper setting of a myopathology laboratory, a preliminary diagnosis of muscle biopsy for treatable myopathies can be reported on the same working day. The result can provide a very fast and reliable diagnosis for the clinicians to start immediate treatment for the patient. I hope this review will shed a little more light on the role of muscle biopsy in the correct diagnosis of neuromuscular patients especially the ones who can benefit from available rapid and appropriate therapeutic management.
